# Supplementary material for: Delayed presentation of breast cancer patients and contributing factors in East Africa: Systematic review and meta-analysis
Source: PLoS One. 2024 Nov 11;19(11):e0309792. doi: 10.1371/journal.pone.0309792 (PMC11554124; doi:10.1371/journal.pone.0309792)
Supplement: S2 File — (DOCX) [file pone.0309792.s002.docx]

Supplementary file 4**:** Newcastle-Ottawa Quality Assessment Scale for cross sectional studies used in the systematic review and meta-analysis 2024.

|  | **Selection** | | | | **Comparability** | **Outcome** | | **Total**  **score** | **Quality rating** |
| --- | --- | --- | --- | --- | --- | --- | --- | --- | --- |
| Study reference | Representativeness s (1) | Sample size (1) | Non-respondents (1) | Ascertainment of the exposure (risk factor) (1) | The subjects in different outcome groups are comparable, based on the study design or analysis. confounding factors are controlled (2) | Assessment of the outcome (2) | Statistical test (1) |  |  |
| Anissa Mohammed Hassen etal,2021 | * | * | * | 0 | * | ** | * | *******(7) | High quality |
| Tesfaw, et al,2020 | * | * | * | * | * | ** | * | ********(8) | High quality |
| Birtukan Shewarega etal,2023 | * | * | * | * | * | ** | * | *********(9) | High quality |
| JabirAbdella Muhammed etal,2022 | * | * | * | * | * | * | * | *******(8) | High quality |
| Mezgebu Abiye etal,2023 | * | * | * | * | * | ** | * | *******(7) | High quality |
| Alem Gebremariam,etal,2019 | * | * | * | ** | * | ** | * | *********(9) | High quality |
| Lydia E. Pace,etal,2015 | * | * | * | * | 0 | ** | * | *******(7) | High quality |
| Alaaddin M Salih,etal  ,etal,2016 | * | * | * | * | * | ** | * | ********(8) | High quality |
