# Supplementary material for: Delayed presentation of breast cancer patients and contributing factors in East Africa: Systematic review and meta-analysis
Source: PLoS One. 2024 Nov 11;19(11):e0309792. doi: 10.1371/journal.pone.0309792 (PMC11554124; doi:10.1371/journal.pone.0309792)
Supplement: S3 File — (DOCX) [file pone.0309792.s003.docx]

**Supplementary S2 file: Searching strategy**

| Google scholar | | | | Result |
| --- | --- | --- | --- | --- |
| Concept | | | Search detail |  |
| Concept1 map | Delayed presentation breast cancer patient | Keyword | " Delayed presentation breast cancer patient  "[tw] OR "late presentation of breast cancer patient"[ tw] OR "breast cancer care"[tw] OR “late stage diagnosis of breast cancer ”[tw] OR “timing of diagnosis breast cancer”[tw] OR “breast cancer”[tw] | 12043 |
|  |  | Mesh term | “delayed breast cancer presentation ”[Mesh] | 351 |
| Concept 3 map | Associated factors | Keyword | "Associated factors" [tw] OR "determinants"[tw] OR "Predictors"[tw] OR "Factors contributing"[tw] OR "correlates"[tw] | 4702 |
| Concept 3 map | Breastcancer patient | Keyword | "breast cancer caret"[tw] “Breastcancer patient”[tw] | 451 |
|  |  | Mesh term | "breast cancer patient"[MeSH Terms] | 780 |
| Concept 4 map | East Africa | Keyword | East Africa [tw] | 541 |
|  |  | Mesh term | "East Africa "[MeSH Terms] |  |
| Concept 5 map | Prevalence | Keywords | Prevalence[tw] or Magnitude[tw] OR Proportion[tw] | 5623 |
|  |  | Mesh term | "Prevalence"[Mesh] |  |
| (("we used Terms like "breast cancer," "associated factors," "predictors," "determinants," "contributing factors," "prevalence," "magnitude," "proportion," "delayed patient presentation," "late presentation breast cancer," "late diagnosis breast cancer," "late diagnosis of patient," "East Africa," In addition, eastern African countries, namely, Ethiopia, Ertriea, Sudan, South Sudan, Djibouti, Kenya, Rwanda, Zimbabwe, Tanzania, Uganda, Somalia, Burundi, Namibia, Botswana, Reunion, Mayotte, Seychelles,Madagaskar, Marituis and Democratic republic of Congo were also included to ensure a comprehensive search. We experimented and improved utilizing several test searches, combining related search phrases with Boolean operators like OR and combining distinct notions using Boolean operator AND. | | | | 2134 |
| PubMed | | | |  |
| Delayed presentation of breast cancer patient in East Africa. | | | | 342 |
| **Other database sources** | | | | 104 |
