# Supplementary material for: Delayed presentation of breast cancer patients and contributing factors in East Africa: Systematic review and meta-analysis
Source: PLoS One. 2024 Nov 11;19(11):e0309792. doi: 10.1371/journal.pone.0309792 (PMC11554124; doi:10.1371/journal.pone.0309792)
Supplement: S4 File — (DOCX) [file pone.0309792.s004.docx]

Supplementary file 1: description of inclusion/exclusion articles

| no | Study reference | Title | Country | Study design | Sample size | Included /excluded | Reasons for exclusion | Link to Unpublished Studies |
| --- | --- | --- | --- | --- | --- | --- | --- | --- |
| 1 | Tesfaw A etal,2020 | Patient Delay and Contributing Factors Among Breast Cancer Patients at Two Cancer Referral Centres in Ethiopia: | Ethiopia | A Cross-Sectional Study | 371 | included | NA | N/A |
| 2 | Tesfaw A etal,2021 | Late-Stage Diagnosis and Associated Factors Among Breast Cancer Patients in South and Southwest Ethiopia: | Ethiopia | Cross-sectional | 426 | excluded | The study did not measure the outcome of interest of delayed presentation of breast cancer. | N/A |
| 3 | Hassen AM etal,2021 | Factors Associated with Delay in Breast Cancer Presentation at the Only Oncology Center in North East Ethiopia | Ethiopia |  | 204 | included | N/A | N/A |
| 4 | Tesfaw A etal,2020 | Why women with breast cancer presented late to health care facility in North-west Ethiopia? A qualitative study | Ethiopia | Qualitative | - | excluded | The study design was not suitable for answering the research question. In case of qualitative study when quantitative data were required. | N/A |
| 5 | Gebremariam A et al,2023 | Association of Delay in Breast Cancer Diagnosis With Survival in Addis Ababa, Ethiopia: | Ethiopia | A Prospective Cohort Study | 439 | Excluded | Outcome not relevant | N/A |
| 6 | Muhammed JA etal,2022 | Prevalence and Factors Associated With Delay in Presentation of Breast Cancer Patients in Ethiopia: | Ethiopia | A Cross-Sectional Institution-Based Study | 150 | Included | N/A | N/A |
| 7 | Dye TD et al,2012 | Experience of Initial Symptoms of Breast Cancer and Triggers for Action in Ethiopia | Ethiopia | Qualitative | 69 | excluded | Because of a qualitative study when quantitative data were required. | N/A |
| 8 | Bedada T etal, 2017 | Prevalence and Factors Contributing to Late Diagnosis of Breast Cancer among Women Attending Tikur Anbessa Specialized Hospital, Oncology Unit, Addis Ababa, Ethiopia, 2017 | Ethiopia | Crossectional study | 215 | excluded | Different outcome definition | N/A |
| 9 | Mekonnen AG etal,2022 | Experience of patients with breast cancer with traditional treatment and healers’ understanding of causes and manifestations of breast cancer in North Shewa zone, Ethiopia: a phenomenological study | Ethiopia | Qualitative | 8 | Excluded | The study design was qualitative and we need quantitative analysis | N/A |
| 10 | Misganaw M,etal,2023 | Mortality rate and predictors among patients with breast cancer at a referral hospital in northwest Ethiopia: A retrospective follow-up study | Ethiopia | Cohort | 456 | excluded | Different outcome definition | N/A |
| 11 | Yoseph R etal,2021 | Retrospective Analysis of Breast Cancer Cases Operated in Jush within Four Years Time Period, Jimma, Ethiopia | Ethiopia | Cross-sectional | 116 | Excluded | The study did not measure the outcome of interest, such as delayed presentation of breast cancer. | N/A |
| 12 | Teshome B etal,2021 | Perceived barriers to timely treatment initiation and social support status among women with breast cancer in Ethiopia | Ethiopia | cross-sectional | 196 | Excluded | The study did not measure the outcome of interest, such as delayed presentation of breast cancer. It focuses on barriers on diagnosis to treatment | N/A |
| 13 | Pace LE etal,2015 | Delays in Breast Cancer Presentation and Diagnosis at Two Rural Cancer Referral Centers in Rwanda | Rwanda | cross-sectional | 144 | Included | N/A | N/A |
| 14 | Areri HA etal,2018 | Survival status and predictors of mortality among Breast Cancer patients in Adult Oncology Unit at Black Lion Specialized Hospital, Addis Ababa, Ethiopia,2018. | Ethiopia | Cohort | 627 | excluded | Different out come definition | N/A |
| 15 | Alem B etal,2022 | Delay in Health-Seeking Behavior and Associated Factors Among Cancer Patients in The Amhara Region Referral Hospitals, Ethiopia. | Ethiopia | cross-sectional | 636 | Excluded | Different outcome variable | N/A |
| 16 | Dedey F etal,2016 | Factors Associated With Waiting Time for Breast Cancer Treatment in a Teaching Hospital in Ghana | Ghana | Cross-sectional | 205 | Excluded | Different out come variable and out of our study area | N/A |
| 17 | Getachew S etal,2020 | Perceived barriers to early diagnosis of breast Cancer in south and southwestern Ethiopia: | Ethiopia | a qualitative study | 27 | Excluded | The study design is qualitative approach. | N/A |
| 18 | Daniel O etal,2023 | Delayed breast cancer presentation, diagnosis, and treatment in Kenya | Kenya | Mixed methods | 378 | Excluded | Out come variable is not relevant and the study lacked essential data needed for analysis of determinant of delayed presentation of breast cancer patient | N/A |
| 19 | Otieno E etal,2010 | Provider delay in the diagnosis and initiation of definitive treatment for breast cancer patients | Kenya | Cross-sectional | 389 | Excluded | Different out come variable | N/A |
| 20 | Salih AM etal,2016 | [Factors Delaying Presentation of Sudanese Breast Cancer Patients: an Analysis Using Andersen's Model](https://journal.waocp.org/article_32360_701fdd24a8a573656c065ea7990b7161.pdf) | Sudan | Cross-sectional | 63 | Included | N/A | N/A |
| 21 | Muddather HF etal,2021 | Survival Outcomes of Breast Cancer in Sudanese Women: A Hospital-Based Study | Sudan | Cohort | 225 | Excluded | Different outcome variable | N/A |
| 22 | Elgoraish A etal,2021 | Patient delay impact on breast cancer survival at Khartoum Referral Hospital: a retrospective study | Sudan | Crossectional | 411 | Excluded | Different outcome variable .it focuses on the impact of delay on breast cancer patient | N/A |
| 23 | Rick TJ etal,2021 | Barriers to Cancer Care in Northern Tanzania: Patient and Health-System Predictors for Delayed Presentation | Tanzania | Cohort | 244 | excluded | The outcome variable is not specific it deals on cancer care | N/A |
| 24 | Mabula JB etal,2012 | Stage at diagnosis, clinicopathological and treatment patterns of breast cancer at Bugando Medical Centre in north-western Tanzania | Tanzania | crossectional | 384 | Excluded | Different outcome variable | N/A |
| 25 | Scheel JR etal,2017 | Breast Cancer Beliefs as Potential Targets for Breast Cancer Awareness Efforts to Decrease Late-Stage Presentation in Uganda | Uganda | cross-sectional | 401 | Excluded | different out interest | N/A |
| 26 | Muchuweti D, etal,2017 | Factors Contributing to Delayed Breast Cancer Presentation: A Prospective Study at Parirenyatwa Group of Hospitals, Harare, Zimbabwe 2010-2013 | Zimbabwe | Crossectional | 73 | Excluded | The study did not provide enough data for meaningful analysis or failed to report key metrics. The results were expressed by simple descriptive not by logistic regression to determine the odd ratio and upper and lower confidence interval | N/A |
| 27 | Ranaivomanana Metal,2019 | Management of Breast Cancer in The Oncology Department of University Hospital of Tambohobe Fianarantsoa | Madagascar | cross-sectional | 52 | Excluded | Different outcome variable | N/A |
| 28 | ABIYE M,etal,2023 | Delay And Contributing Factors In Patients With Breast Cancer At Hiwot Fana Comprehensive Specialized Hospital, Harar, Eastern Ethiopia: Haramaya University Harar; | Ethiopia | Cross-sectional | 206 | Included | N/A | N/A |
| 29 | Anakwenze C etal,2018 | Factors Related to Advanced Stage of Cancer Presentation in Botswana | Botswana | cross-sectional study | 214 | Excluded | Different out come variable and not specific about breast cancer | N/A |
