# Supplementary material for: Delayed presentation of breast cancer patients and contributing factors in East Africa: Systematic review and meta-analysis
Source: PLoS One. 2024 Nov 11;19(11):e0309792. doi: 10.1371/journal.pone.0309792 (PMC11554124; doi:10.1371/journal.pone.0309792)
Supplement: S5 File — (DOCX) [file pone.0309792.s005.docx]

Supplementary file 2:data extraction of included articles in the systematic review and meta- analysis

| Study reference | Name of Data Extractor(s) | Date of Data Extraction | Eligibility Confirmation | Reason for Inclusion | | Extracted Data |
| --- | --- | --- | --- | --- | --- | --- |
| Anissa Mohammed Hassen etal, 2021 | Tadele Emagneneh,Assefa Sisay | March 10,2024 | Yes | Met all inclusion criteria. | | Publication date:2021  Study design: Cross-sectional  Country: Ethiopia  sample size :204  effect size :50.5(43.6, 57.4)  Outcome measures: Breast cancer |
| Aragaw Tesfaw etal, 2020 | Assefa Sisay ,Getinet Kumie | March 15,2024 | Yes | Met all inclusion criteria. | | Publication date:2020  Study design :Cross-sectional  Country: Ethiopia  sample size : 371  effect size : 75.7(71.3, 80)  Outcome measures: Breast cancer |
| Birtukan Shewaregaetal, 2023 | Abebaw Alamrew,Chalie Mulugeta | March 17 | Yes | Met all inclusion criteria. | | Publication date:2023  Study design: Cross-sectional  Country: Ethiopia  sample size: 269  effect size: 67(62.1, 71.7)  Outcome measures: Breast cancer |
| Jabir Abdella Muhammedetal, 2022 | Betelhem Ejigu ,Getinet Kumie | March 19,2024 | Yes | Met all inclusion criteria. | | Publication date:2022  Study design :Cross-sectional  Country: Ethiopia  sample size : 150  effect size : 57.3(51.3, 63)  Outcome measures: Breast cancer |
| Alem Gebremariametal, 2019 | Betelhem Ejigu ,Chalie Mulugeta | March 20,2024 | Yes | Met all inclusion criteria. | | Publication date:2019  Study design :Cross-sectional  Country: Ethiopia  sample size : 441  effect size : 36 (33, 38.7)  Outcome measures: Breast cancer |
| Mezgebu Abiye etal, 2023 | Abebaw Alamrew,Tadele Emagneneh | March 13,2024 | Yes | Met all inclusion criteria. | | Publication date:2023  Study design :Cross-sectional  Country: Ethiopia  sample size : 206  effect size : 76.7(70.8, 82.6)  Outcome measures: Breast cancer |
| LydiaE. Pace etal, 2015 | Assefa Sisay,Abebaw Alamrew | March 07,2024 | Yes | Met all inclusion criteria. | | Publication date:2015  Study design :Cross-sectional  Country: Rwanda  sample size : 144  effect size : 58 (51.9, 64.1)  Outcome measures: Breast cancer |
| AlaaddinM Salih,etal  Etal, 2016 | Chalie Mulugeta ,Getinet Kumie | March 29,2024 | Yes | | Met all inclusion criteria. | Publication date:2016  Study design :Cross-sectional  Country: Sudan  sample size : 63  effect size : 74.6 (64.1, 85)  Outcome measures: Breast cancer |
