# Supplementary material for: Delayed presentation of breast cancer patients and contributing factors in East Africa: Systematic review and meta-analysis
Source: PLoS One. 2024 Nov 11;19(11):e0309792. doi: 10.1371/journal.pone.0309792 (PMC11554124; doi:10.1371/journal.pone.0309792)
Supplement: S6 File — (DOCX) [file pone.0309792.s006.docx]

Supplementary file 3: characteristics of the included articles

| **no** | **Author** | **Year** | **Study Design** | **Country** | **Sample size** | **Frequency** | **Outcome Measures** | **ES**[95%C]** | **Additional variable** | **Source of additional data** | **Date of additional data obtained** | |
| --- | --- | --- | --- | --- | --- | --- | --- | --- | --- | --- | --- | --- |
| 1 | Anissa Mohammed Hassen,etal | 2021 | Cross-sectional | Ethiopia | 204 | 102 | Breast cancer patient | 50.5(43.6, 57.4) | age ≥40  socioeconomic status  breast pain  awareness of breast cancer, visit traditional healer | N/A | N/A |  |
| 2 | Aragaw Tesfaw,etal | 2020 | Cross-sectional | Ethiopia | 371 | 280 | Breast cancer patient | 75.7(71.3, 80) | Socioeconomic status, breast pain, lumpunderarmpit, having comorbidity | N/A | N/A |  |
| 3 | Birtukan Shewarega,etal | 2023 | Cross-sectional | Ethiopia | 269 | 180 | Breast cancer patient | 67(62.1, 71.7) | Socioeconomic status, residence,  no awareness of breast CA, lack of money | N/A | N/A |  |
| 4 | Jabir Abdella Muhammed,etal | 2022 | Cross-sectional | Ethiopia | 150 | 86 | Breast cancer patient | 57.3(51.3, 63) | Educational status, employee  no breast pain  visit traditional healer,  no family hx of breast cancer | N/A | N/A |  |
| 5 | Alem Gebremariam,etal | 2019 | Cross-sectional | Ethiopia | 441 | 159 | Breast cancer patient | 36 (33, 38.7) | Age, educational status, marital status,parity,awarnes of breast cance,pain of breast cancer | N/A | N/A |  |
| 6 | Mezgebu Abiye,etal | 2023 | Cross-sectional | Ethiopia | 206 | 157 | Breast cancer patient | 76.7(70.8, 82.6) | Educational status, residence. Breast pain ,visit traditional healer, distance from home to health institution | N/A | N/A |  |
| 7 | LydiaE. Pace,etal | 2015 | Cross-sectional | Rwanda | 144 | 84 | Breast cancer patient | 58 (51.9, 64.1) | Age, educational status, marital status, awareness of breast cancer, breast pain, visit traditional healer, family history of breast cancer, having comorbidity | N/A | N/A |  |
| 8 | AlaaddinM Salih,etal  ,etal | 2016 | Cross-sectional | Sudan | 63 | 47 | Breast cancer patient | 74.6 (64.1, 85) | Family history of breast cancer, awareness of breast cancer, marital status,employe.residence,educational status ,age | N/A | N/A |  |
