# Supplementary material for: Delayed presentation of breast cancer patients and contributing factors in East Africa: Systematic review and meta-analysis
Source: PLoS One. 2024 Nov 11;19(11):e0309792. doi: 10.1371/journal.pone.0309792 (PMC11554124; doi:10.1371/journal.pone.0309792)
Supplement: Attachment — Submitted filename: Response to Reviewers.docx [file pone.0309792.s007.docx]

**Reply to the reviewers’ comments**

We would like to thank you for your time, comments and suggestions for improving the manuscript. The responses to the reviewers' comments and suggestions have been highlighted in blue and the main document of the manuscript in yellow for better assessment. Point by point responses for academic editor and the four reviewers are listed below.

**Responses to academic editor**

1. Please ensure that your manuscript meets PLOS ONE's style requirements, including those for file naming.

Dear academic editor thank you for your valuable comments based on that we write the manuscript based on PLOS ONE guidelines.

2. Please amend your list of authors on the manuscript to ensure that each author is linked to an affiliation. Authors’ affiliations should reflect the institution where the work was done (if authors moved subsequently, you can also list the new affiliation stating “current affiliation:….” as necessary).

Based on your comment we correctly write author affiliation and list of authors (page 1 and 2, line number 6 and 29-42).

3. We note that your Data Availability Statement is currently as follows: [All relevant data are within the manuscript and its Supporting Information files.]

Thank you dear editor we accept your comment and rewrite it in the manuscript (page 21,line number 513).

**Response to reviewers**

Reviewer #1

1) Does the study cover all countries in East Africa or is it limited to certain countries?

Dear reviewer thank you for your constructive comments. The study covers all countries in East Africa mentioned in the method section of searching strategy (page 8).

2) Does the study include the most recent data or is it limited to a specific time period?

Thank you dear reviewer for your insights. The study includes all data available up to the point of the research. However, it focuses recent data to ensure the data's without time boundary. This approach allows for a comprehensive analysis of the current situation regarding delayed breast cancer presentation in East Africa. For further information it was stated in the method section (page 6,line number 155).

3) Did the study consider the impact of socioeconomic status on the behaviour of delayed medical seeking?

Yes, we were considering socioeconomic status affects medical seeking behavior. In the previous articles mentioned in the introduction section low socioeconomic status(education level, income and occupation) were positively associated with the delay in the medical seeking behavior(page 5,line number 123 - 125). In this study specifically analyzed low education level status was significantly associated with delays in seeking medical care(page 14,line number316). However, employee patients were not statically significant with delayed medical seeking behavior (page15 ,line number 354)

4) Did the study examine the effect of access to health care on delayed medical consultation?

Yes, we were examining the effect of access to health care on delayed medical consultation. From the introduction section accessibility of health facility, proximity to healthcare facilities, availability of healthcare professionals, and affordability of services were positively associated with delay for medical consultation(page 5,line number124) In this meta- analysis distance >5km from home to health facility were one of the contributing factors of delayed the medical consultation(page 15,line number 336).

5) Did the study analyse the impact of culture and beliefs on the behaviour of delayed medical consultation? Yes, from previous articles mentioned in the background section. It examined various cultural factors such as traditional healing practices, cultural beliefs, and community norms were positive impact on the delay presentation of breast cancer patients(page 5,line number 123 -125). In our meta-analysis visit traditional healer of breast cancer patient were positive impact with delay for medical consultation (page 15,line number 344).

6) Did the study consider the impact of the diagnostic capabilities of different health care facilities on delayed diagnosis?

Thank you dear reviewer for your comment. The objective of this Meta -analysis mainly focuses on delayed patient presentation (delayed in health seeking behavior).The study did not specifically address the impact of the diagnostic capabilities of healthcare facilities on delayed diagnosis. The focus was primarily on other factors such as patient behavior and socioeconomic status.

However, as supportive availability of diagnostic equipment, the training level of medical staff, and the overall capacity of facilities to perform accurate and timely diagnoses were mentioned in the introduction section.

7) Did the study assess the impact of patients' knowledge about breast cancer on their behaviour in seeking medical care?

We try to analysis and search literatures to investigate factors such as awareness of breast cancer symptoms, understanding of risk factors, and knowledge of early detection methods were written in the background section (page 4and5,line number110-114). In this Meta -analysis awareness of breast cancer was not significant factor with delayed presentation of breast cancer patient (page 16, line number363).
8) Does the study consider the impact of patients' self-perception of symptoms on their health care seeking behaviour?

Yes, we write patients recognized and interpreted their symptoms and their level of concern about these symptoms based on their perceptions in the back ground section (page 4 and 5).

In this meta-analysis the findings indicated that patients who perceived there is no lump under armpit were not associated with delay health seeking behavior (page 16 ,line number369).
9) The manuscript should be proofread for grammatical errors, spelling mistakes or unclear presentation.

Thank you Dear reviewer based on your comments the manuscript were checked grammar error, rephrased, reworded and corrected spelling errors.
10) The methods section should be more precise, detailed and scientific.

Based on your comments the method section were revised and more précised and specific to the topic (page5-9).

**Reviewer#2: General Comments**:
1.AbstractClarityandConciseness:
o The abstract should be more concise and clearly highlight the key findings and their implications. It currently includes repetitive information and lacks a clear structure.

Thank you Dear reviewer based on your constructive comments the introduction section of abstract were reworded and rewrite in structured way(page 3,line number 54-58).
The results section in the abstract should provide specific statistics on delay prevalence and the most significant factors contributing to the delay, rather than a general overview.

Based on your suggestion the result section of the abstract were rephrased, written clearly and included the most important significant factors(page 3,line number 66-72).

2. Introduction:
o The introduction section is lengthy and contains excessive background information. Condense it to focus more on the rationale for the study, specific research questions, and the study's significance.
Thank you Dear reviewer based on that the background section was including the rational of the study, specific research questions and the significance of the study. We were rephrased and the length of the background was minimized in to two pages (page 4 and 5).

The rational of the study was clearly written in the last paragraph (page 5,line number 127-140).

o Some of the statistics and details can be moved to a background subsection if necessary.
Thank you dear reviewer we try to condense similar concepts in one paragraph (page 4 and 5).

3. Methods:
o Search Strategy: The search strategy section is comprehensive but lacks clarity in describing the inclusion and exclusion criteria. Consider using a table to summarize these criteria for better readability.
Based on your comment the inclusion and exclusion criteria were summarized by table (page 7, line number173).

Table 1: inclusion and exclusion criteria

| **Study characteristics** | **Inclusion criteria** | **Exclusion criteria** |
| --- | --- | --- |
| Design | observational studies Cross-sectional studies | Clinical trials, qualitative studies, editorial letters, case report/series |
| Population | Breast cancer patients | Patients with malignancies of other body parts |
| Condition | Delayed presentation of breast cancer patients | Unclear to determine time of presentation of breast cancer pateint , articles only reviews and descriptive static’s |
| Context | Studies conducted in East Africa | Studies not from East Africa |
| Language | English | English |

o Quality Assessment: More detail is needed on how the Newcastle-Ottawa Scale was applied and the criteria used for assessing the quality of included studies.

To ensure the reliability and validity of the findings, the quality of the included studies was assessed using the Newcastle-Ottawa Scale (NOS). The NOS is a tool used to evaluate the quality of non-randomized studies in meta-analyses. It includes three broad perspectives: the selection of study groups, the comparability of the groups, and the ascertainment of either the exposure or outcome of interest for case-control or cohort studies, respectively. However, our included articles were cross-sectional.

A table summarizing the quality scores of the included studies would be helpful.
We accept your comment and based on that we prepare a summarizing table for the quality scores of the included studies and used as supplementary material (page 9,line number 220).

**Supplementary S 3 file :** Newcastle-Ottawa Quality Assessment Scale for cross sectional studies used in the systematic review and meta-analysis 2024

|  | **Selection** | | | | **Comparability** | **Outcome** | | **Total score** |
| --- | --- | --- | --- | --- | --- | --- | --- | --- |
| Authors | Representativeness s (1) | Sample size (1) | Non respondents (1) | Ascertainment of the exposure (risk factor) (2) | The subjects in different outcome groups are comparable, based on the study design or analysis. confounding factors are controlled (1) | Assessment of the outcome (2) | Statistical test (1) |  |
| Anissa Mohammed Hassen,etal. | 1 | 1 | 1 | 0 | 1 | 2 | 1 | 7 |
| Tesfaw, et al. | 1 | 1 | 1 | 1 | 1 | 2 | 1 | 8 |
| Birtukan Shewarega,etal. | 1 | 1 | 1 | 2 | 1 | 2 | 1 | 9 |
| JabirAbdella Muhammed,etal. | 1 | 1 | 1 | 2 | 1 | 1 | 1 | 8 |
| Mezgebu Abiye,etal. | 1 | 1 | 1 | 0 | 1 | 2 | 1 | 7 |
| Alem Gebremariam,etal. | 1 | 1 | 1 | 2 | 1 | 2 | 1 | 9 |
| Lydia E. Pace,etal. | 1 | 1 | 1 | 1 | 0 | 2 | 1 | 7 |
| Alaaddin M Salih,etal  ,etal. | 1 | 1 | 1 | 1 | 1 | 2 | 1 | 8 |

4. Results:
o The results section should present the key findings more clearly, with distinct subsections for different aspects of the study (e.g., prevalence of delayed presentation, factors contributing to the delay).

Based on your insight we write the result section with distinct subsection; the prevalence of delayed presentation breast cancer and contributing factors of delayed presentation of breast cancer in to subheadings (page12 and 13).

5. Discussion:
o The discussion should provide a deeper analysis of the results, comparing them with findings from other regions or previous studies.

Thank you dears based on your feedback we discussed the discussion part deeply by comparing other regions and countries (page17-19,line number387-471).

Discuss the implications of the high prevalence of delayed presentation and the identified factors in more detail.

We try to write the burden of high prevalence delayed presentation of breast cancer and contributing factors in east Africa region(page 17,line number 387-395).
o Highlight the strengths and limitations of the study more explicitly.

We write the strength and limitation of the study clearly in separate way (page 20, line number 475-489)

Discuss how the limitations might have affected the results and suggest areas for future research.

Thank you dear reviewer based on your comment we explain the implication of the limitation on the manuscript and suggestions for future research (page 20,line number 484-487).

6. Conclusion:
o The conclusion should be more focused on summarizing the key findings and their implications for public health policies and interventions in East Africa.

Based on your comment the conclusion part were written in clear way and recommend healthcare stakeholder and policy makers (page 20 ,line number 491-499).
o Avoid introducing new information in the conclusion section.

We accept your comment. Based on that we the conclusion effectively summarizes the key findings and their implications for public health without adding new details or insights.

7. Overall Language and Style:
o The manuscript would benefit from a thorough proofreading to correct grammatical errors and improve the overall readability. Some sentences are overly complex and could be simplified.

Thank you dear reviewer based on your comments the manuscript checked by proofreading to correct grammar and spelling error.

**Specific Comments:**
1. Abstract:
o "The overall analysis of delay presentation breast cancer patient was 61.85%..." should be rephrased for clarity.

Thank you dear reviewer we rephrase and rewrite it like this the pooled prevalence of delayed presentation breast cancer patient in East Africa was 61.85% (95% Confidence Interval: 48.83 – 74.88%)(page 3,line number 66).

o Results should be summarized with specific data points and concise explanations.

Based on your comment we write the result in short and clear way (page3,line number 66-72).

2. Introduction:
o Condense the first two paragraphs to avoid redundancy. Focus on the unique context of East Africa.

Based on your suggestion the introduction part were rephrased and making in one with similar concepts (page 4,line number 82-89)

The last paragraph of the introduction should clearly state the study objectives and research questions.
Based on your comment edit and rewrite the last paragraph (page 5,line number 127-140).

3.Methods

o Clarify the search terms and databases used. Consider presenting the search strategy in a table.
Dear reviwer based on your comment we prepare searching terms by using table as supplementary S 2 file(page 8, line number 189).

**Searching strategy additional file**

| Google scholar | | | | Result |
| --- | --- | --- | --- | --- |
| Concept | | | Search detail |  |
| Concept1 map | Delayed presentation breast cancer patient | Keyword | " Delayed presentation breast cancer patient  "[tw] OR "late presentation of breast cancer patient"[ tw] OR "breast cancer care"[tw] OR “late stage diagnosis of breast cancer ”[tw] OR “timing of diagnosis breast cancer”[tw] OR “breast cancer”[tw] | 12043 |
|  |  | Mesh term | “delayed breast cancer presentation ”[Mesh] | 351 |
| Concept 3 map | Associated factors | Keyword | "Associated factors" [tw] OR "determinants"[tw] OR "Predictors"[tw] OR "Factors contributing"[tw] OR "correlates"[tw] | 4702 |
| Concept 3 map | Breastcancer patient | Keyword | "breast cancer caret"[tw] “Breastcancer patient”[tw] | 451 |
|  |  | Mesh term | "breast cancer patient"[MeSH Terms] | 780 |
| Concept 4 map | East Africa | Keyword | East Africa [tw] | 541 |
|  |  | Mesh term | "East Africa "[MeSH Terms] |  |
| Concept 5 map | Prevalence | Keywords | Prevalence[tw] or Magnitude[tw] OR Proportion[tw] | 5623 |
|  |  | Mesh term | "Prevalence"[Mesh] |  |
| (("we used Terms like "breast cancer," "associated factors," "predictors," "determinants," "contributing factors," "prevalence," "magnitude," "proportion," "delayed patient presentation," "late presentation breast cancer," "late diagnosis breast cancer," "late diagnosis of patient," "East Africa," In addition, eastern African countries, namely, Ethiopia, Ertriea, Sudan, South Sudan, Djibouti, Kenya, Rwanda, Zimbabwe, Tanzania, Uganda, Somalia, Burundi, Namibia, Botswana, Reunion, Mayotte, Seychelles,Madagaskar, Marituis and Democratic republic of Congo were also included to ensure a comprehensive search. We experimented and improved utilizing several test searches, combining related search phrases with Boolean operators like OR and combining distinct notions using Boolean operator AND. | | | | 2134 |
| PubMed | | | |  |
| Delayed presentation of breast cancer patient in East Africa. | | | | 342 |
| **Other database sources** | | | | 104 |

Provide a rationale for the selection of studies based on the Newcastle-Ottawa Scale

Newcastle-Ottawa Scale is one of the quality assessment tools. Here is the rationale for using the NOS and how it guided the selection process: Comprehensive Assessment of Study quality, ensuring methodological rigor and focus on relevant studies.

4. Results:
o The PRISMA flow diagram should be included to illustrate the study selection process.

Based on your suggestion we elaborate the PRISMA flow diagram to select our included articles. It was elaborated like this

**Identification of studies via database**

**Records identified from database**
 PubMed=100

- Hinari=10
- Cochrane Review=85
- EMBASE=10
- Google Scholar=895

**Total=900**

**Recorded removed before screening**

- Duplicate record=200
- Recorded by other reasons=0

**Identification**

Recorded screened=900

Title/ abstract excluded=100

Methodological issue=125

Not focused on east Africa=250

Not relevant to breast cancer=325

**Screening**

Reports assessed for eligibility= 100

Different outcome definition=60

Overlapping of participants=7

Lack of full data=15

Did not meet quality criteria=10

**Studies included in the review=8**

**Included**

Fig1: PRISMA flowchart diagram of the study selection process

o Present the findings related to each factor contributing to the delay in separate subsections or a summary table.

Based on your comment we summarized the associated factors by table(page 13,line number 305).

| **Variable** | **Exposed** | **Comparator** | **OR (95% CI)** | **I 2** |
| --- | --- | --- | --- | --- |
| Visit Traditional medicine healer | Yes | No | 3.52; (1.43, 5.59). | 99.4% |
| Age | >40 | <40 | 1.87; (1.03, 2.71). | 99% |
| Educational status | Illiterate | Literate | 3.61 ;( 2.39, 4.82). | 98.3% |
| Residence | Rural | Urban | 3.33;( 2.16, 4.49). | 98.6% |
| Distance from home to health facility | >5km | <5km | 2.89; (1.54, 4.24). | 97.5% |
| Breast Pain | Not feeling breast pain | Feeling breast pain | 2.42;( 1.09, 3.74) | 99% |

5. Discussion:
o Compare the study’s findings with those of similar studies from other regions.

Thank you dears based on your insight we explain the discussion part with other regions (page 17-19).
o Discuss potential reasons for the high prevalence of delayed presentation in East Africa.

Based on tour comments we were explained the high prevalence of delayed breast cancer patient in east Africa region (page 17,line number 387-395).

o Highlight the strengths and limitations of the study more explicitly.

Based on your comment we were write the strength and limitation of the study in this analysis (page 20, line number 475-487).

Discuss how the limitations might have affected the results and suggest areas for future research.

Thank you dear reviewer based on your comment we explain the implication of the limitation on the manuscript and suggestions for future research (page 20,line number 484-487).

One of the limitations of the study is the fact that recall bias may not have been completely eliminated from the study, as almost all studies included in the study were cross-sectional in nature and it is possible that the outcome variable was under- or overestimated. The high heterogeneity indicates that the studies are not suffiecntly similar to combine meaningfully, leading potentially misleading conclusion. Studies that were published only in the English language were taken into account.

We suggest for future researcher inclusion of diverse study design, focus on subgroup analysis and improved stastical techniques for handling heterogeneity and missing data, it is better to take a qualitative approach and include articles published by different language (20, line number 484-490).

6. Conclusion:
o The conclusion should be more focused on summarizing the key findings and their implications for public health policies and interventions in East Africa.

Thank you dear reviwer.Based on your comments the conclusion part was rewrite and focused on the specific findings (page 20, line number 491-498).

o Avoid introducing new information in the conclusion section.

We accept your comment. Based on that we the conclusion effectively summarizes the key findings and their implications for public health without adding new details or insights.

**Reviewer #3: Reviewer Comments:**

1. The introduction effectively contextualizes the significance of the study within the broader global health landscape. It clearly articulates the urgency of addressing late presentations of breast cancer in East Africa, which is essential for guiding future interventions and policies.

Thank you for your positive feedback.

2.The authors adhered to the PRISMA guidelines meticulously, ensuring transparency and reproducibility in their systematic review and meta-analysis.

Thank you dear reviewer for your comments.

The search strategy was robust, encompassing multiple databases and employing comprehensive search terms relevant to the study objectives.

Thank you.

3.The analysis of data regarding the prevalence of late patient presentations and associated factors was thorough and well-documented.

The inclusion of quality assessment criteria and the justification for study selection criteria added to the credibility of the findings.

Thank you dears the analysis of this meta-analysis were written clearly by dividing sub in to sections and for more the quality assessment tool were documented and summarizing by table(page 7,line number 173).

4.The discussion effectively synthesizes the findings, providing insights into the implications for breast cancer management and public health strategies in East Africa.

Thank you Dear reviewer. For further explanation the discussion part was written deeply by comparing the result with other regions(page 17-19).

It appropriately discusses the limitations of the study and suggests avenues for future research

Thank you dear reviewer for your positive feedback. The limitation of the study was discussed with its implication on this analysis and we suggest future researcher(page20,line number 479-489).

5. The character of the included studies needs to be described in detail.

Thank you dears based on your comment the significant factors associated with delayed presentation of breast cancer were written clearly by comparing previous literature (page 18 and 19).

**Reviewer #4:** 
We need to know if there any updated data after 2020 in Background section.
Dear reviewer based on your comment we include resent data about late presentation of breast cancer the introduction section (page 4 and 5).
And it is necessary to explain the heterogeneity in the Discussion section.

Thank you dear reviewer based on your constructive comments we discussed the heterogeneity and subgroup analysis in the discussion part(page 18,line number 414-423).

--------------------------------- **Thank you**!!!!------------------------------------
