# Supplementary material for: Delayed presentation of breast cancer patients and contributing factors in East Africa: Systematic review and meta-analysis
Source: PLoS One. 2024 Nov 11;19(11):e0309792. doi: 10.1371/journal.pone.0309792 (PMC11554124; doi:10.1371/journal.pone.0309792)
Supplement: Attachment — Submitted filename: Response to Reviewer.docx [file pone.0309792.s008.docx]

**Reply to the reviewers’ comments**

We would like to thank you for your time, comments and suggestions for improving the manuscript. The responses to the reviewers' comments and suggestions have been highlighted in blue and the main document of the manuscript in track changes for better assessment. Point by point responses for academic editor and the one reviewer are listed below.

**Response to Academic Editor comments:**

Please review your reference list to ensure that it is complete and correct. If you have cited papers that have been retracted, please include the rationale for doing so in the manuscript text, or remove these references and replace them with relevant current references. Any changes to the reference list should be mentioned in the rebuttal letter that accompanies your revised manuscript. If you need to cite a retracted article, indicate the article’s retracted status in the References list and also include a citation and full reference for the retraction notice.

Dear Editor,

Thank you for your detailed review and suggestions regarding the reference list in our manuscript. In response to your request, we have carefully reviewed the entire reference list to ensure that it is complete, accurate, and up-to-date.

**Review of Reference List:** We have thoroughly checked each reference and confirmed that all citations are correct, including author names, publication details, and other necessary information.

**Retracted References:** After conducting a comprehensive search, we did not find any retracted papers among the references cited in our manuscript.

**Confirmation of Accuracy:** We are confident that the reference list is complete and all references are accurate and relevant to our study.

Thank you for your continued guidance. We hope that these confirmations meet your requirements and contribute to the integrity of our manuscript.

**Response to Reviewer comments:**

Reviewer #2: Overall Evaluation
The paper provides a comprehensive analysis of the potential for using mRNA expression data to predict biochemical recurrence (BCR) in prostate cancer (PCa) patients pre-operatively. The use of machine learning methodologies to model time-to-event data is innovative and demonstrates significant improvements in predictive performance compared to traditional clinical models. The study is well-structured and the methodology is sound, however, there are several areas where the paper could benefit from additional clarification and minor revisions.
Major Strengths
1. Innovative Approach: The use of mRNA expression data pre-operatively for BCR prediction in PCa is novel and holds promise for improving patient outcomes.
2. Comprehensive Methodology: The paper employs a range of machine learning models and provides a thorough evaluation of their performance using multiple metrics.
3. Clinical Relevance: The focus on pre-operative prediction aligns well with the clinical need for early and accurate decision-making in prostate cancer treatment.
Minor Revisions
1. Clarification of Calibration Methodology:
o The paper describes two forms of calibration analyses but the explanation of the calibration curves and how they were derived is somewhat dense. Consider breaking down the steps more clearly, perhaps with additional visual aids or flowcharts to guide the reader through the process.
o Specifically, provide more detailed explanations on how the Kaplan-Meier estimates were used in the calibration curves and the rationale behind using the quintiles.
2. Detailed Explanation of Feature Selection:
o While the study discusses the feature selection rates, there is limited discussion on why certain mRNA variables were selected over others. A deeper analysis of the biological relevance of the frequently selected mRNA variables (e.g., DNAH8, ABCC11, ESM1) and their known roles in PCa or other cancers would strengthen the discussion.
o It would be beneficial to include a table summarizing the key mRNA variables, their known functions, and any previous associations with cancer to provide context to their selection.
3. Discussion of Model Limitations:
o The paper acknowledges the modest cohort size and single-centre data source as limitations. It would be useful to discuss potential biases that might arise from this and how they might impact the generalizability of the results.
o Consider elaborating on how multi-centre validation could address these limitations and the specific steps that will be taken in future research to ensure robustness and applicability across diverse populations.
4. Figure Improvements:
o Figures 1 and 2 are crucial for understanding the calibration performance of the models but could benefit from higher resolution and clearer labels. Ensure that all axes and legends are easy to read.
o In Figure 5, it would be helpful to add more context or annotations to highlight key observations regarding the expression levels of mRNA variables in patients receiving neoadjunctive therapy versus those who did not.
5. ROC and DCA Analysis:
o The ROC and DCA analyses are well-presented but could be further enhanced by including confidence intervals for the AUC values in the ROC plots directly, rather than just in the table. This visual representation would make it easier to compare the performance across models.
o In the DCA plots, consider adding a brief explanation of how net benefit is interpreted and its clinical implications, as some readers may not be familiar with this analysis.
6. Discussion on Clinical Implementation:
o The conclusion mentions the potential for clinical integration of these models but does not elaborate on the practical steps required to achieve this. Provide more details on what would be needed for these models to be adopted in a clinical setting, including any regulatory considerations, necessary validation studies, and potential barriers to implementation.
Conclusion
This paper presents a significant contribution to the field of prostate cancer research by demonstrating the potential of mRNA-based pre-operative prediction models. With minor revisions to enhance clarity, provide deeper biological insights, and discuss implementation strategies, this study could offer valuable guidance for the development of precision medicine tools in oncology.

**Response to Reviewer #2:**

Dear Reviewer,

Thank you for your thoughtful review and the detailed feedback provided. However, it appears that there has been a mix-up, and the comments you provided do not correspond to the manuscript titled "Delayed Presentation of Breast Cancer Patients and Associated Factors in east Africa." The review comments seem to refer to a different paper that focuses on mRNA expression data for predicting biochemical recurrence in prostate cancer patients. The model of the review seems machine learning, however our research model is systematic review and meta-analysis. Our research focuses on understanding the factors contributing to the delayed presentation of breast cancer, which is critical for improving early detection and patient outcomes. The analysis, methodology, and conclusions presented in the manuscript are directly related to breast cancer, not prostate cancer or mRNA expression data.

Given this discrepancy, we kindly request that you review our manuscript titled "Delayed Presentation of Breast Cancer Patients and Associated Factors" and provide feedback relevant to the content of our study. We believe that your insights and expertise will be help in improving the quality of our research.

We look forward to receiving your revised feedback and are eager to address any concerns or suggestions you may have related to our study on delayed presentation of breast cancer patients and associated factors.

Thank you for your understanding and attention to this matter.
